# APOSTEL 2.0 Recommendations for Reporting Quantitative Optical Coherence Tomography Studies

**DOI:** 10.1212/WNL.0000000000012125

**Published:** 2021-07-13

**Authors:** Aykut Aytulun, Andrés Cruz-Herranz, Orhan Aktas, Laura J. Balcer, Lisanne Balk, Piero Barboni, Augusto Azuara Blanco, Peter A. Calabresi, Fiona Costello, Bernardo Sanchez-Dalmau, Delia Cabrera DeBuc, Nicolas Feltgen, Robert P. Finger, Jette Lautrup Frederiksen, Elliot Frohman, Teresa Frohman, David Garway-Heath, Iñigo Gabilondo, Jennifer S. Graves, Ari J. Green, Hans-Peter Hartung, Joachim Havla, Frank G. Holz, Jaime Imitola, Rachel Kenney, Alexander Klistorner, Benjamin Knier, Thomas Korn, Scott Kolbe, Julia Krämer, Wolf A. Lagrèze, Letizia Leocani, Oliver Maier, Elena H. Martínez-Lapiscina, Sven Meuth, Olivier Outteryck, Friedemann Paul, Axel Petzold, Gorm Pihl-Jensen, Jana Lizrova Preiningerova, Gema Rebolleda, Marius Ringelstein, Shiv Saidha, Sven Schippling, Joel S. Schuman, Robert C. Sergott, Ahmed Toosy, Pablo Villoslada, Sebastian Wolf, E. Ann Yeh, Patrick Yu-Wai-Man, Hanna G. Zimmermann, Alexander U. Brandt, Philipp Albrecht

**Affiliations:** From the Department of Neurology, Medical Faculty (A.A., O.A., H.-P.H., O.M., S.M., M.R., P.A.), Heinrich-Heine University Düsseldorf, Germany; Department of Neurology (A.C.-H., A.J.G.), University of California San Francisco; Departments of Neurology, Population Health, and Ophthalmology (L.J.B., R.K.), NYU Grossman School of Medicine, New York, NY; Mulier Institute (L.B.), Centre for Research on Sports in Society, Utrecht, the Netherlands; Scientific Institute San Raffaele (P.B.), Milan, Italy; Centre for Public Health (A.A.B.), Queen's University Belfast, Northern Ireland, UK; Division of Neuroimmunology (P.A.C., S. Saidha), Johns Hopkins University, Baltimore, MD; Departments of Clinical Neurosciences and Surgery (F.C.), University of Calgary, Alberta, Canada; Institut d’Investigacións Biomediques August Pi iSunyer (IDIBAPS) and Hospital Clinic (B.S.-D., E.H.M.-L., P.V.), University of Barcelona, Spain; Bascom Palmer Eye Institute (D.C.D.), University of Miami Miller School of Medicine, FL; Department of Ophthalmology (N.F.), University Medical Center, Göttingen; Department of Ophthalmology (R.P.F., F.G.H.), University of Bonn, Germany; Department of Neurology (J.L.F., G.P.-J.), Rigshospitalet Glostrup and University of Copenhagen, Denmark; Laboratory of Neuroimmunology (E.F., T.F.), Stanford University School of Medicine, CA; Institute of Ophthalmology (D.G.-H.), National Institute for Health Research (NIHR) Biomedical Research Centre at Moorfields Eye Hospital NHS Foundation Trust and UCL Institute of Ophthalmology (D.G.-H.), London, UK; Biocruces Bizkaia Health Research Institute (I.G.), Barakaldo, Spain; Department of Neurosciences (J.S.G.), University of California, San Diego; Brain and Mind Centre (H.-P.H.), University of Sydney, Australia; Department of Neurology (H.-P.H.), Medical University of Vienna, Austria; Institute of Clinical Neuroimmunology (J.H.), LMU Hospital, Ludwig-Maximilians Universität München, Germany; UConn Health Comprehensive MS Center, Division of Multiple Sclerosis and Neuroimmunology, Department of Neurology (J.I.), University of Connecticut School of Medicine, Farmington; Faculty of Medicine and Health Sciences (A.K.), Macquarie University, Sydney, Australia; Department of Neurology (B.K., T.K.), Klinikum rechts der Isar, School of Medicine, Technical University of Munich, Germany; Department of Medicine and Radiology (S.K.), University of Melbourne, Australia; Department of Neurology with Institute of Translational Neurology (J.K.), University of Münster; Eye Center, Medical Center, Faculty of Medicine (W.A.L.), University of Freiburg, Germany; Experimental Neurophysiology Unit (L.L.), Institute of Experimental Neurology (INSPE), IRCCS San Raffaele, University Vita-Salute San Raffaele, Milan, Italy; Lille Neurosciences & Cognition (O.O.), Univ Lille, Inserm, CHU Lille, U1172-LilNCog (JPARC), France; Experimental and Clinical Research Center (F.P., H.G.Z., A.U.B.), Max Delbrück Center for Molecular Medicine and Charité-Universitätsmedizin Berlin, corporate member of Freie Universität Berlin, Humboldt-Universität zu Berlin, and Berlin Institute of Health, Germany; Moorfields Eye Hospital (A.P.), The National Hospital for Neurology and Neurosurgery, Queen Square, UCL Institute of Neurology, London, UK; Neuro-ophthalmology Expert Center (A.P.), Amsterdam UMC, the Netherlands; Department of Neurology, First Faculty of Medicine (J.L.P.), Charles University and General University Hospital in Prague, Czech Republic; Department of Ophthalmology (G.R.), Ramon y Cajal Hospital, Medicine University of Alcalá, Madrid, Spain; Department of Neurology (M.R.), Center for Neurology and Neuropsychiatry, LVR-Klinikum, Heinrich-Heine-University Düsseldorf, Germany; Department of Neurology (S. Schippling), University Hospital Zurich, Switzerland; Departments of Ophthalmology, Neuroscience, and Physiology (J.S.S.), NYU Langone Health, NYU Grossman School of Medicine, New York; Departments of Biomedical Engineering, Electrical and Computer Engineering (J.S.S.), NYU Tandon School of Engineering, Brooklyn, NY; Thomas Jefferson University Medical College (R.C.S.), Philadelphia, PA; Queen Square MS Centre, Department of Neuroinflammation (A.T.), UCL Institute of Neurology, University College London, UK; Departments of Ophthalmology and Clinical Research (S.W.), Bern University Hospital, University of Bern, Switzerland; Division of Neurology, Department of Pediatrics (E.A.Y.), Hospital for Sick Children, Division of Neurosciences and Mental Health SickKids Research Institute, University of Toronto, Canada; Department of Clinical Neurosciences (P.Y.-W.-M.), University of Cambridge; Moorfields Eye Hospital (P.Y.-W.-M.), London, UK; University of California (A.U.B.), Irvine; and IMSVISUAL (A.A., A.C.-H., O.A., L.J.B., L.B., P.A.C., F.C., J.L.F., E.F., T.F., I.G., J.S.G., A.J.G., H.-P.H., J.H., J.I., R.K., A.K., B.K., T.K., J.K., L.L., E.H.M.-L., S.M., O.O., F.P., A.P., G.P.-J., J.L.P., M.R., S. Saidha, S. Schippling, R.C.S., P.V., E.A.Y., H.G.Z., A.U.B., P.A.), International Multiple Sclerosis Visual System Consortium, Middleton, WI.

## Abstract

**Objective:**

To update the consensus recommendations for reporting of quantitative optical coherence tomography (OCT) study results, thus revising the previously published Advised Protocol for OCT Study Terminology and Elements (APOSTEL) recommendations.

**Methods:**

To identify studies reporting quantitative OCT results, we performed a PubMed search for the terms “quantitative” and “optical coherence tomography” from 2015 to 2017. Corresponding authors of the identified publications were invited to provide feedback on the initial APOSTEL recommendations via online surveys following the principle of a modified Delphi method. The results were evaluated and discussed by a panel of experts and changes to the initial recommendations were proposed. A final survey was recirculated among the corresponding authors to obtain a majority vote on the proposed changes.

**Results:**

A total of 116 authors participated in the surveys, resulting in 15 suggestions, of which 12 were finally accepted and incorporated into an updated 9-point checklist. We harmonized the nomenclature of the outer retinal layers, added the exact area of measurement to the description of volume scans, and suggested reporting device-specific features. We advised to address potential bias in manual segmentation or manual correction of segmentation errors. References to specific reporting guidelines and room light conditions were removed. The participants' consensus with the recommendations increased from 80% for the previous APOSTEL version to greater than 90%.

**Conclusions:**

The modified Delphi method resulted in an expert-led guideline (evidence Class III; Grading of Recommendations, Assessment, Development and Evaluations [GRADE] criteria) concerning study protocol, acquisition device, acquisition settings, scanning protocol, funduscopic imaging, postacquisition data selection, postacquisition analysis, nomenclature and abbreviations, and statistical approach. It will be essential to update these recommendations to new research and practices regularly.

Increases in the numbers of quantitative optical coherence tomography (OCT) studies have raised the need for consistent and coherent standardized reporting recommendations. In 2016, the Advised Protocol for OCT Study Terminology and Elements (APOSTEL) recommendations were published to provide a 9-point checklist of relevant aspects for reporting quantitative retinal OCT studies.^1^ The original APOSTEL recommendations were conceived as expert opinion (level D evidence according to the Grading of Recommendations Assessment, Development and Evaluation [GRADE] working group criteria; gradeworkinggroup.org) from discussions among the authors, the IMSVISUAL consortium (imsvisual.org), and consideration of the literature.^2^ Without a formal consensus-building approach, and without involving a broader audience, further validation was warranted. We aimed to revise and achieve consensus on these recommendations by using a modified Delphi method, including a larger group of OCT scientists and clinicians, in a formal procedure to review the consensus and develop level C evidence-based guidelines (GRADE criteria).^3^ The long-term goal was to improve the reproducibility and interoperability of OCT studies for retinal and neuro-ophthalmology diseases.

## Methods

**Figure 1:**
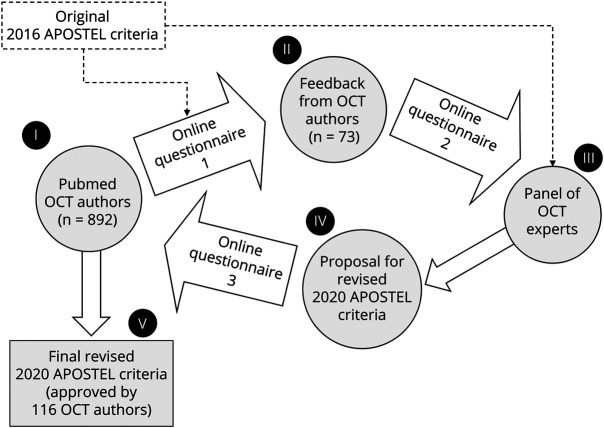
Modified Delphi Method The modified Delphi method is described as a consensus-building process. We contacted 892 authors of quantitative (optical coherence tomography [OCT]) studies identified by PubMed (I) using an online survey, in which feedback on the original Advised Protocol for OCT Study Terminology and Elements (APOSTEL) 2016 criteria was requested. The feedback of the 73 responding OCT authors was analyzed by a panel of experts (II) and changes to the APOSTEL recommendations were proposed (III). A revised version (IV) was proposed to the OCT authors (n = 116), who approved the revisions by majority vote, which led to the final revised 2020 APOSTEL criteria (V).

In order to identify experts in the field while minimizing the risk of selection bias, we chose to contact corresponding authors of studies reporting quantitative retinal OCT results published within 24 months prior to our initial survey by email. A total of 892 authors of 1,189 publications were identified by a PubMed search (performed 3 July 2017) using the search terms “quantitative” and “optical coherence tomography” for 2015 to 2017. The Delphi method is a systematic, multistage survey to obtain consensus on a specified question. The process involves multiple rounds of questionnaires presented to participants. The responses are analyzed by a panel of experts and fed back to participants and assessed for consensus.^4^ Most of the members of the panel of experts were also corresponding authors of quantitative retinal OCT studies and were therefore also invited to participate in the survey. Following the consensus-building procedure of a modified Delphi method (figure 1), we conducted the following steps:

1. We contacted all corresponding authors of the identified publications and asked them to evaluate and give feedback on the initial APOSTEL recommendations. The participants were asked about their agreement on each item of the recommendations, rating from 1 (full disagreement) to 4 (full approval). Participants were given the opportunity to provide comments. In a blinded fashion, we collected feedback and suggestions using a free online survey via Google Forms (initial questionnaire; raw data of survey results can be obtained from the corresponding author upon qualified request).
2. We then formed a panel of 54 international experts who gathered at congress meetings and during 4 rounds of telephone conferences. The aggregated results of the initial questionnaire were reviewed online through a second questionnaire by the panel, who also revised the original APOSTEL recommendations and proposed a list of changes.
3. This list was then reviewed in a second Delphi round by the original group of corresponding authors through a third online questionnaire (Google Forms). In this last Delphi round, the participants were given the opportunity to approve or reject the final list of suggestions of the panel of experts by majority vote.

## Results

### Initial Questionnaire: Survey About the Initial APOSTEL Recommendations Among Corresponding Authors

Seventy-three (8%) of the 892 contacted corresponding authors of quantitative OCT studies completed the first online questionnaire and provided feedback, the majority of these being ophthalmologists (71%), followed by neurologists (10%) and neuro-ophthalmologists (10%). Eighty percent of participants agreed with the recommendations as they were published and 95% planned to adhere to the recommendations in future publications. At the same time, 64% stated having reported their previous research with less detail than suggested.

### Second Questionnaire: Consensus Building With the Panel of Experts

Based on the feedback obtained during the first survey, the panel of 54 experts drafted a list of 15 suggested changes to the original APOSTEL recommendations. Twelve (80%) of these suggestions (see below) were accepted through the second questionnaire, while proposals already covered in the original recommendations or to include OCT angiography (OCT-A) were rejected. With this feedback, we generated a revised version of the APOSTEL recommendations with an updated 9-point checklist.

### Third Questionnaire: Second Delphi Round With Corresponding Authors

A total of 116 (13%) of the 892 corresponding authors responded to the third survey. Among them, 53% were ophthalmologists, 35% neurologists, and 12% non-MD researchers. The overall acceptance of the proposed changes was over 95%, with the only exception of the recommendation to report the pixel to millimeter ratio and the image format if the images are exported from the device for analysis, which was accepted by 84% of the authors.

### Summary of Revisions

**Figure 2:**
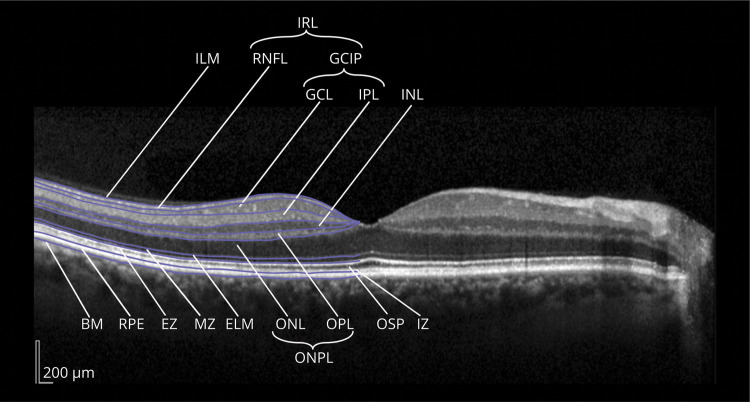
Consensus Nomenclature for Retinal Structures The different layers (and their boundaries) are illustrated in a central horizontal spectral-domain optical coherence tomography scan through the middle of the fovea. Retinal structures and layers: BM = Bruch membrane; ELM = external limiting membrane; EZ = ellipsoid zone (inner and outer segment junction); GCL = ganglion cell layer; ILM = inner limiting membrane; INL = inner nuclear layer; IPL = inner plexiform layer; IZ = interdigitation zone; MZ = myoid zone; ONL = outer nuclear layer; OPL = outer plexiform layer; OSP = outer segment of the photoreceptors; RNFL = retinal nerve fiber layer; RPE = retinal pigment epithelium. Compound layers: GCIP = ganglion cell and inner plexiform layer (composite of macular GCL and IPL); IRL = inner retinal layers (composite of macular RNFL, GCL, and IPL); ONPL = outer nuclear and plexiform layer (composite of ONL and OPL). Copyright by IMSVISUAL and licensed under CC-BY-4.0 for this publication (imsvisual.org/resources/media).

After the modified Delphi process for consensus building, we decided to maintain the initial recommendations of stating the acquisition protocol and imaging modalities and addressing concomitant eye pathologies with the exact scanning protocol. The changes made to the original APOSTEL recommendations checklist are highlighted in the table and summarized below:

1. As already addressed in correspondence to the initial recommendations,^5^ we harmonized the nomenclature of the outer retinal layers to match the 2014 consensus article by Staurenghi et al.^6^ (figure 2).
2. We removed references to specific reporting guidelines to avoid favoring any guidelines or omitting relevant recommendations.
3. When utilized, we suggest reporting device-specific features (e.g., enhanced depth imaging, swept-source OCT, adaptive optics).
4. We added the exact area of measurement (e.g., analysis grids) to the description of volume scans.
5. We added a commentary regarding the importance of addressing potential bias in manual segmentation or manual correction of segmentation errors (masking). In several comments, concerns were raised regarding the length of the methodology section of articles that fully adhered to the APOSTEL recommendations. In case of limited word count availability, we now advise submitting the exact OCT methodology as supplementary material, if permitted.
6. Another issue raised by several comments was concerning the relevance of some of the details to be reported regarding the acquisition setting, namely the room lighting conditions and whether pupils were dilated. The panel of experts agreed that reporting the ambient lighting condition is likely to be of low clinical importance, although shaded room lighting is suggested. However, off-axis beam placement could affect the results of OCT imaging studies, and the risk for this phenomenon increases with pupil dilation and is greater for the outer retinal layers (outer plexiform layer/outer nuclear layer) compared to the inner retinal layers (peripapillary retinal nerve fiber layer to inner nuclear layer).^7^ Oberwahrenbrock and colleagues^8^ showed that the greatest error is for the outer retinal layers. Therefore, pupil dilation is relevant because it can directly affect quantitative OCT measures. We thus omitted room light conditions but retained pupil dilation.

**Table:**
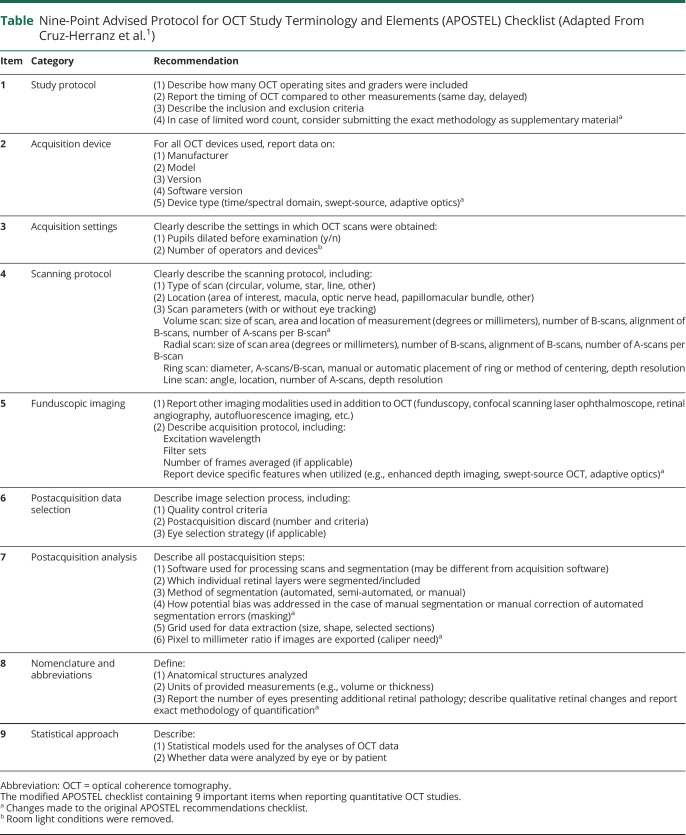
Nine-Point Advised Protocol for OCT Study Terminology and Elements (APOSTEL) Checklist (Adapted From Cruz-Herranz et al.^1^)

## Discussion

The formal consensus-building approach of a modified Delphi method was used to revise the APOSTEL recommendations for the reporting of quantitative OCT studies.

We observed a high consensus of the participants already with the initial APOSTEL recommendations in the first survey. The majority of the participants acknowledged the need for guidance.

Whereas the original APOSTEL recommendations were conceived by a panel dominated by neurologists, a more heterogeneous mix of specialties, with broader expertise, contributed to this new version, the majority being ophthalmologists. Ninety-seven percent of all participants agreed that that the APOSTEL 2.0 guidelines should apply to all studies reporting on quantitative retinal OCT research and not be restrained to certain disorders or disciplines. Furthermore, choosing to identify the experts to be addressed by the survey as the corresponding authors of relevant research articles based on a PubMed search assured a broad consensus-building approach, eliminating the selection bias typically immanent to expert consortia. However, there was a low response rate^9^: 8% of the contacted corresponding authors responded to the first round of the survey and 13% to the second round. Possible explanations for this limitation may include the fact that corresponding authors are senior supervisors or principal investigators and are not necessarily as involved in the technical details and specifications addressed by the APOSTEL recommendations. Likewise, there are time constraints to consider. This can be viewed as a limitation of the study but we have to assume that those who participated in the survey were knowledgeable about the matter and contact details for the first authors or technicians involved in these studies were not available.

The modified Delphi method tends to eliminate extreme (but possibly relevant) positions and steers a middle-course consensus. However, all survey participants were given the opportunity to provide feedback in free text and all comments were critically discussed among the panel of experts. The achieved consensus is based on the opinion of the participants and the panel of experts and therefore it should be regularly counterchecked and revised along with evolving scientific evidence.

These recommendations do not cover all aspects and techniques possibly amenable to OCT research and are based on expert opinion and a single consensus finding investigation rather than on a systematic review of a large body of literature. Therefore, they are not intended as an indispensable premise for all experimental OCT research. The APOSTEL recommendations are intended for clinical OCT studies using established techniques and help to provide the necessary comparability between studies.

Some additions suggested during the revision process were not included in the final version as consensus was not reached. One of these suggestions was to incorporate a section on OCT-A. However, the inclusion of details pertaining to OCT-A in the APOSTEL 2.0 recommendations would be premature. The field of OCT-A, both clinically and academically, is in a phase of rapid evolution and essentially in its infancy. Its use is not well established in routine clinical care in either the fields of ophthalmology or neurology. Interpretation of OCT-A scans across devices is challenging and standardized quantitative OCT-A metrics are lacking or vary across OCT platforms. Moreover, there is a lack of consensus regarding quality control criteria for image acquisition and the implementation of such standards as they pertain to OCT-A. These limitations are likely to change in the future. For these reasons, the evidence and corresponding investigative and clinical recommendations for OCT and OCT-A should remain on separate tracks.

A future revision of the APOSTEL criteria likely will also need to consider the role of artificial intelligence–based data from image analyses.^10^

We present revised APOSTEL recommendations based on this investigation using a modified Delphi process that involves a broad group of experts. Therefore, the resulting APOSTEL 2.0 can be considered an expert-led guideline (evidence class C, GRADE criteria) covering all relevant aspects of quantitative retinal OCT research. It will be necessary to update these recommendations to new research and practices regularly.

## Glossary

APOSTEL: Advised Protocol for OCT Study Terminology and Elements
GRADE: Grading of Recommendations Assessment, Development and Evaluation
OCT: optical coherence tomography
OCT-A: optical coherence tomography angiography
